# Circulating tumor DNA dynamics and recurrence risk in patients undergoing curative intent resection of colorectal cancer liver metastases: A prospective cohort study

**DOI:** 10.1371/journal.pmed.1003620

**Published:** 2021-05-03

**Authors:** Jeanne Tie, Yuxuan Wang, Joshua Cohen, Lu Li, Wei Hong, Michael Christie, Hui Li Wong, Suzanne Kosmider, Rachel Wong, Benjamin Thomson, Julian Choi, Adrian Fox, Kathryn Field, Matthew Burge, Jenny Shannon, Dusan Kotasek, Niall C. Tebbutt, Christos Karapetis, Craig Underhill, Andrew Haydon, Joy Schaeffer, Janine Ptak, Cristian Tomasetti, Nicholas Papadopoulos, Kenneth W. Kinzler, Bert Vogelstein, Peter Gibbs

**Affiliations:** 1 The Walter and Eliza Hall Institute of Medical Research, Melbourne, Australia; 2 Western Health, Melbourne, Australia; 3 Peter MacCallum Cancer Centre, Melbourne, Australia; 4 Faculty of Medicine, Dentistry and Health Sciences, University of Melbourne, Australia; 5 Sidney Kimmel Comprehensive Cancer Center, Johns Hopkins Medicine, Baltimore, Maryland, United States of America; 6 Royal Melbourne Hospital, Melbourne, Australia; 7 Eastern Health, Melbourne, Australia; 8 Eastern Health Clinical School, Faculty of Medicine, Nursing and Health Sciences, Monash University, Melbourne, Australia; 9 Royal Brisbane and Women’s Hospital, Brisbane, Australia; 10 Nepean Cancer Care Centre, Sydney, Australia; 11 Icon Cancer Centre, Adelaide, Australia; 12 Olivia Newton-John Cancer and Wellness Centre, Melbourne, Australia; 13 Flinders Medical Centre, Flinders University, Adelaide, Australia; 14 Border Medical Oncology, Albury, Australia; 15 The Alfred Hospital, Melbourne, Australia; 16 Johns Hopkins Bloomberg School of Public Health, Baltimore, Maryland, United States of America

## Abstract

**Background:**

In patients with resectable colorectal liver metastases (CRLM), the role of pre- and postoperative systemic therapy continues to be debated. Previous studies have shown that circulating tumor DNA (ctDNA) analysis, as a marker of minimal residual disease, is a powerful prognostic factor in patients with nonmetastatic colorectal cancer (CRC). Serial analysis of ctDNA in patients with resectable CRLM could inform the optimal use of perioperative chemotherapy. Here, we performed a validation study to confirm the prognostic impact of postoperative ctDNA in resectable CRLM observed in a previous discovery study.

**Methods and findings:**

We prospectively collected plasma samples from patients with resectable CRLM, including presurgical and postsurgical samples, serial samples during any pre- or postoperative chemotherapy, and serial samples in follow-up. Via targeted sequencing of 15 genes commonly mutated in CRC, we identified at least 1 somatic mutation in each patient’s tumor. We then designed a personalized assay to assess 1 mutation in plasma samples using the Safe-SeqS assay. A total of 380 plasma samples from 54 patients recruited from July 2011 to Dec 2014 were included in our analysis. Twenty-three (43%) patients received neoadjuvant chemotherapy, and 42 patients (78%) received adjuvant chemotherapy after surgery. Median follow-up was 51 months (interquartile range, 31 to 60 months). At least 1 somatic mutation was identified in all patients’ tumor tissue. ctDNA was detectable in 46/54 (85%) patients prior to any treatment and 12/49 (24%) patients after surgery. There was a median 40.93-fold (19.10 to 87.73, *P* < 0.001) decrease in ctDNA mutant allele fraction with neoadjuvant chemotherapy, but ctDNA clearance during neoadjuvant chemotherapy was not associated with a better recurrence-free survival (RFS). Patients with detectable postoperative ctDNA experienced a significantly lower RFS (HR 6.3; 95% CI 2.58 to 15.2; *P* < 0.001) and overall survival (HR 4.2; 95% CI 1.5 to 11.8; *P* < 0.001) compared to patients with undetectable ctDNA. For the 11 patients with detectable postoperative ctDNA who had serial ctDNA sampling during adjuvant chemotherapy, ctDNA clearance was observed in 3 patients, 2 of whom remained disease-free. All 8 patients with persistently detectable ctDNA after adjuvant chemotherapy have recurred. End-of-treatment (surgery +/− adjuvant chemotherapy) ctDNA detection was associated with a 5-year RFS of 0% compared to 75.6% for patients with an undetectable end-of-treatment ctDNA (HR 14.9; 95% CI 4.94 to 44.7; *P* < 0.001). Key limitations of the study include the small sample size and the potential for false-positive findings with multiple hypothesis testing.

**Conclusions:**

We confirmed the prognostic impact of postsurgery and posttreatment ctDNA in patients with resected CRLM. The potential utility of serial ctDNA analysis during adjuvant chemotherapy as an early marker of treatment efficacy was also demonstrated. Further studies are required to define how to optimally integrate ctDNA analyses into decision-making regarding the use and timing of adjuvant therapy for resectable CRLM.

**Trial registration:**

ACTRN12612000345886.

## Author summary

**Why was this study done?:**

- Recurrence risk remains high in patients who underwent curative-intent surgical resection of colorectal cancer liver metastases (CRLM), with limited additional benefit from pre- or postoperative chemotherapy.
- There is currently no validated biomarker of recurrence for resected CRLM that could help guide the use of chemotherapy for the individual patient.
- Circulating tumor DNA (ctDNA), representing DNA shed from tumor cells into the circulation, is a versatile blood-based biomarker that can provide real-time assessment of cancer burden.
- It has been shown in a previous small study of 18 patients that ctDNA detection after surgery for CRLM is highly predictive of eventual cancer relapse.

**What did the researchers do and find?:**

- We performed a larger study involving 54 patients with resectable CRLM to confirm the ability of postoperative ctDNA to detect microscopic residual disease and predict relapse. We also analysed serial ctDNA during and after chemotherapy. ctDNA was detected in 24% of patients immediately after surgery, and these patients had a very high recurrence risk of 83% compared to only 31% in those with undetectable ctDNA after surgery.
- All patients with detectable postoperative ctDNA who failed to clear their ctDNA following adjuvant chemotherapy experienced recurrence, while 67% of patients whose ctDNA became undetectable after chemotherapy remained disease-free.

**What do these findings mean?:**

- ctDNA detection after surgery or after completion of adjuvant chemotherapy is associated with a very high risk of recurrence and death in patients with resectable CRLM; ctDNA dynamics before and after adjuvant chemotherapy reflected adjuvant treatment efficacy.
- Future studies should aim to assess how best to incorporate ctDNA testing into clinical practice to guide adjuvant treatment decision.

## Introduction

The liver is the most common site of metastatic disease in patients with colorectal cancer (CRC) [1]. For the 20% to 30% of metastatic CRC patients with liver-limited metastases, an increasingly aggressive approach to management is being pursued, with the intent of cure [2]. However, recurrence rates following definitive resection of colorectal cancer liver metastases (CRLM) remain high, with limited benefit from perioperative or adjuvant chemotherapy demonstrated in prospective randomised studies [3–6]. Despite this, the use of systemic chemotherapy along with resection of CRLM remains an accepted standard of care [7]. Currently, there are no validated biomarkers of patient recurrence risk that could inform and personalize the optimal use of pre- and/or postsurgery chemotherapy and patient surveillance.

Circulating tumor DNA (ctDNA), representing tumor-specific DNA mutations that can be detected in the cell-free component of the peripheral blood, is a relatively novel and versatile biomarker with potential clinical applications that include noninvasive real-time molecular characterization of tumors [8–10] and real-time assessment of tumor bulk [11–13]. The possibility that detection of ctDNA following curative intent surgery for metastatic CRC could predict recurrence was suggested in an initial series of 18 patients [14]. Of 16 instances where ctDNA was detected postoperatively, 15 (93.8%) had developed recurrence, whereas no recurrences (0%) were observed in patients where ctDNA was not detected.

Here, we report findings of a validation study to confirm the prognostic impact of postoperative ctDNA in resectable CRLM observed in the previous discovery study [14], with analysis extended to include a sample at diagnosis and serial samples taken during any neoadjuvant therapy or adjuvant therapy and then during routine follow-up. The primary objective was to confirm the prognostic impact of postoperative ctDNA on recurrence-free survival (RFS). Secondary objectives included the correlation of ctDNA at diagnosis and at end of treatment, along with ctDNA dynamics during neoadjuvant and adjuvant chemotherapy, with clinical outcomes.

## Methods

### Patients and sample acquisition

This prospective multicentre cohort study recruited patients with upfront resectable CRLM (as determined by the multidisciplinary team) at 10 Australian hospitals (ACTRN12612000345886). The primary tumor had to be either previously resected or deemed to be resectable in the case of synchronous liver metastases. Eligible patients underwent standard staging investigations at diagnosis of liver metastases, including FDG-PET scan, CT scan of chest, abdomen and pelvis, and liver MRI. Patients with extrahepatic metastases or a second malignancy within the last 5 years were excluded.

**Fig 1:**
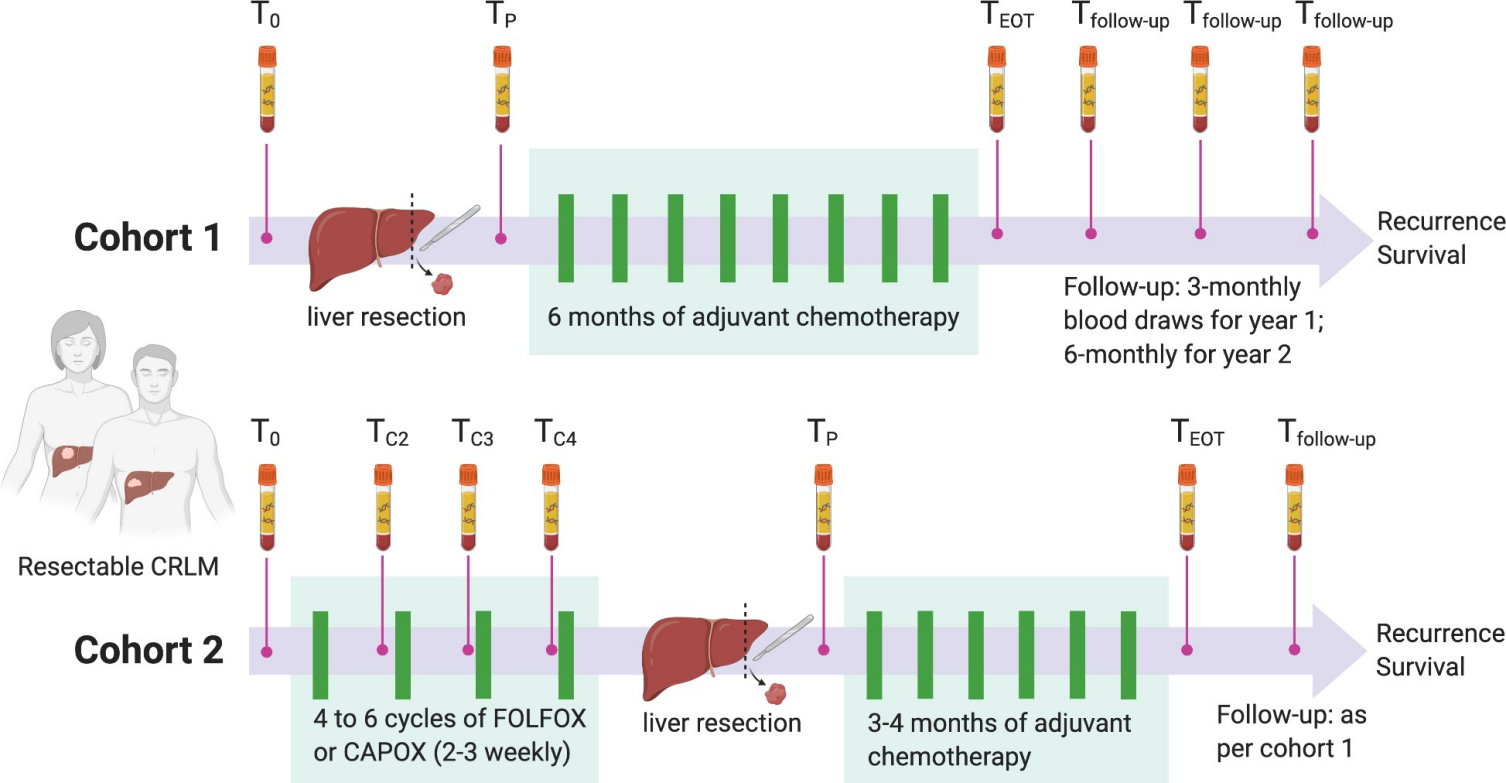
Study design. Study schema showing blood collection time points in Cohort 1 (upfront liver resection) and Cohort 2 (neoadjuvant chemotherapy). The primary objective of the study was to assess the prognostic impact of postoperative ctDNA (T_P_) on recurrence-free survival in the total population. T_0_ = baseline, T_C2_ = pre-cycle 2, T_C3_ = pre-cycle 3, T_C4_ = pre-cycle 4, T_P_ = 4 to 10 weeks postoperative, T_EOT_ = end of treatment, T_follow-up_ = follow-up. CRLM, colorectal cancer liver metastasis; ctDNA, circulating tumor DNA.

The study design and details of blood collection times are shown in Fig 1. Given that both perioperative (pre- and postoperative) and adjuvant (postoperative) chemotherapy approaches are both considered current standard of care, patients were enrolled into 2 cohorts based on clinician’s intent to administer chemotherapy prior to liver resection. Patients planned for upfront liver resection followed by adjuvant chemotherapy were enrolled into cohort 1; patients planned for 4 to 6 cycles of neoadjuvant oxaliplatin-based combination chemotherapy prior to liver resection were enrolled into cohort 2. Following liver resection, the use of up to 6 months (cohort 1) or 3 to 4 months (cohort 2) of adjuvant chemotherapy was recommended. Blood samples for ctDNA and CEA (carcinoembryonic antigen) analysis were collected prior to liver resection (baseline ctDNA, T_0_), prior to each cycle of neoadjuvant chemotherapy for cohort 2 (T_C2_, T_C3_, T_C4_), 4 to 10 weeks after removal of all primary tumor and metastases (postoperative ctDNA, T_P_), at the end of adjuvant chemotherapy (T_EOT_), and during follow-up (every 3 months for year 1 and every 6 months for year 2). At each collection time point, at least 30 mL of blood was drawn into EDTA tubes, centrifuged twice at 1,200*g* and 1,800*g*, and plasma was then stored at −80°C for ctDNA analysis.

Per protocol follow-up after liver resection included clinical review and CEA check every 3 months, with CT imaging every 6 months for 2 years. Thereafter, follow-up was according to the participating institution’s standard of care. Serum CEAs were measured by the diagnostic laboratory at each participating site, with a CEA level of <5 μg/L considered as normal. Pathology reports from liver resection specimens were reviewed to assess known pathologic prognostic factors such as the number of liver metastases and the diameter of the largest liver metastasis. DNA from plasma and tumor samples were purified and analyzed at the Ludwig Center at Johns Hopkins Medical Institutions. The study was approved by the human research ethics committees at each contributing Australian hospital and all participants provided written informed consent. This study is reported as per the Reporting recommendations for tumor marker prognostic studies (REMARK) guideline (S1 REMARK Checklist) [15].

### Circulating tumor DNA analysis

We used a tumor-informed personalized approach for ctDNA analysis, where at least 1 somatic mutation was first identified by targeted sequencing of each patient’s tumor tissue, and the presence of same mutation was then assessed in the plasma samples.

#### Tumor tissue mutation analysis

Formalin-fixed paraffin-embedded tumor tissue from the resected liver metastasis or primary tumor (where tumor tissue from liver metastasis was not available or inadequate for analysis) were analysed for somatic mutations in 15 genes recurrently mutated in CRC (*SMAD4*, *TP53*, *AKT1*, *APC*, *BRAF*, *CTNNB1*, *ERBB3*, *FBXW7*, *HRAS*, *KRAS*, *NRAS*, *PIK3CA*, *PPP2R1A*, *RNF43*, *POLE*). Tumor sections were macrodissected under a dissecting microscope to ensure a neoplastic cellularity of >30%. DNA was purified with a Qiagen FFPE Kit (Qiagen cat #56494). Primers were designed and sequencing results analyzed as previously described [16].

#### Plasma sample mutation analysis

For each patient, the mutation identified in the tumor tissue with the highest mutant allele frequency was assessed in cell-free DNA (cfDNA) from the plasma. The detection and quantitation of ctDNA were performed using the Safe-Sequencing (Safe-SeqS) assay, an error reduction technology for the detection of low frequency mutations, which has been described in detail previously [16–18]. Leukocyte DNA was used to exclude constitutional polymorphisms. ctDNA was classified as detectable (ctDNA-positive) or undetectable (ctDNA-negative) based on a permutation test that compared the mutation frequency in the sample of interest with the mutation frequencies in controls [16]. ctDNA is quantified as mutant allele fraction (MAF), defined as the ratio between the number of “supermutants” (a mutation present in >90% of reads in a unique identifier family with the same molecular barcode) and the number of unique identifier sequences that contain the normal (wild-type) form at the nucleotide of interest. All ctDNA analysis was performed by the study scientists (YW, JC, and BV) blinded to the clinical outcome.

### Statistical analysis

The primary objective of this study was to assess the impact of postoperative ctDNA detection and RFS in the total population (both cohorts combined), which included locoregional and distant recurrence, where deaths without recurrence were censored at the time of death. Based on the initial 18 patient study [14], 13 of 14 (93%) patients who were ctDNA positive after treatment developed recurrence over approximately 2 years, compared to 0 of 4 patients who were ctDNA negative. Among 9 patients who received chemotherapy during the study, 3 (33%) were ctDNA negative after treatment. Using Fisher exact test, a sample size of 25 (allowing for 20% dropout rate) provides >80% power at the <5% significance level to detect at least a 70% difference of RFS at 2 years among ctDNA-positive (20%) compared to ctDNA-negative patients (90%), assuming a 2:1 ratio of ctDNA-positive to ctDNA-negative patients. However, given our secondary aim of exploring the correlation of ctDNA dynamics during neoadjuvant chemotherapy (Cohort 2) and adjuvant chemotherapy (Cohort 1 and 2) with clinical outcomes, a larger sample size of 100 was planned. Due to difficulties in recruitment, study enrolment was ceased after 3.5 years when 61 patients had been recruited. The final analysis of all study endpoints is planned when all patients have been followed up for at least 36 months after liver resection. It is estimated that 80% of recurrences would have occurred at this time point. A prespecified statistical analysis plan is not available for this observational study. However, the analytical approach corresponded to the approach outlined in the study protocol before study data collection and analysis.

Baseline characteristics were compared using the K-sample equality-of-medians test with continuity correction and the two-sample test of proportions. RFS was compared using univariate and multivariate Cox proportional-hazards models, with the exact partial-likelihood method to handle tied event times and adjusting for postoperative ctDNA (negative versus positive), baseline CEA (not elevated versus elevated), number of liver metastases (1 versus >1), diameter of largest liver metastasis (≤3 cm versus >3 cm), time interval from diagnosis of primary tumor to liver metastases (≤12 months versus >12 months), and primary tumour N stage (N0 versus N+) as prespecified confounders. When some groups had no RFS events, thereby resulting in infinite hazard ratios, RFS was instead compared using the two-sample test for difference in survival at 60 months described by Klein and colleagues [19], with log-transformed survival functions and unpooled variances. Although comparisons of baseline characteristics and prespecified confounders were only exploratory in nature, we provided Bonferroni-adjusted significance thresholds of *P* = 0.05 divided by the number of comparisons to assist with interpretation.

Longitudinal MAF measurements were analysed using mixed-effects regression of log-transformed MAF with heteroscedasticity-consistent standard error estimators. Zero-valued MAFs were log-transformed using an offset of 1E–6. The random-intercepts model was chosen over the random-slopes model using the Akaike information criterion [20]. All statistical analyses were conducted using Stata version 15.1 (StataCorp, College Station, United States of America) and R version 3.6.3 (R Foundation for Statistical Computing, Vienna, Austria). In the interests of reproducible research, our raw data and analysis code for the RFS and survival analysis will be available at https://github.com/whong24601/ctDNA2020.

## Results

### Clinicopathological characteristics, pre- and postoperative ctDNA

**Fig 2:**
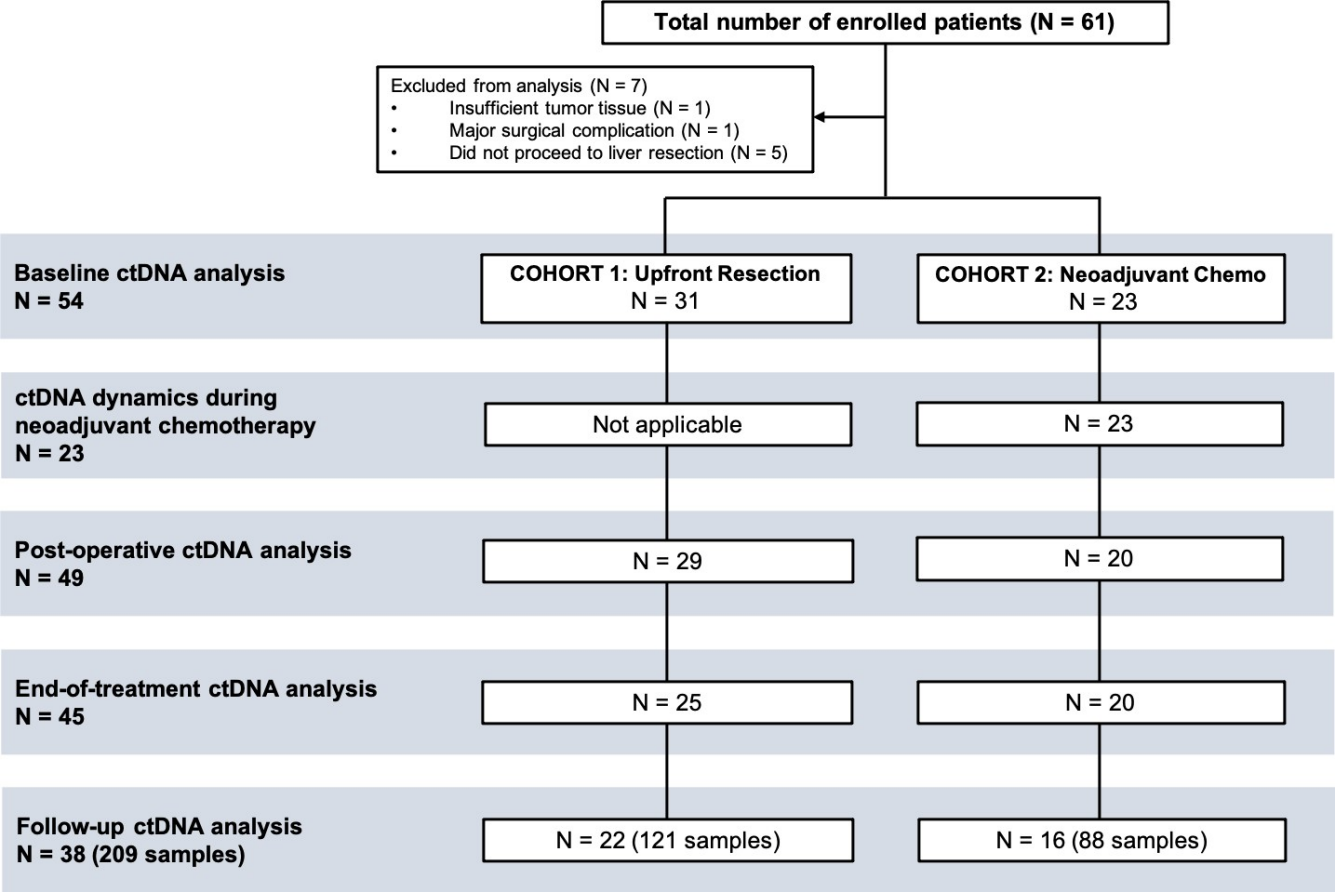
Patient enrolment and samples included in the analyses. Of the 61 enrolled patients, a total of 54 were included in the analysis. Five of these 54 patients who had a baseline blood draw did not have blood collection postoperatively.

We enrolled 61 patients between July 2011 and December 2014. Patient enrolment and plasma samples included in the analysis are presented in Fig 2. Seven patients were excluded from further analysis due to failure to proceed to liver resection (*N =* 5), insufficient tumor tissue for mutation testing (*N* = 1), or a major surgical complication precluding follow-up (*N* = 1).

Clinicopathologic characteristics for the 54 patients included in the analysis are shown in Table 1. The median patient age was 64 years, 70% were male, 46% had synchronous disease, and 39% had more than 1 liver metastasis. The baseline CEA was elevated in 29 patients (54%). The primary tumor had been resected prior to liver resection in all but one patient. Twenty-three patients received upfront chemotherapy. An R0 liver resection was achieved in 30 of the 31 (97%) patients in Cohort 1 and 19 of the 23 (83%) patients in Cohort 2. Forty-two patients (78%) received chemotherapy after surgery (26 from cohort 1 and 16 from cohort 2). Median follow-up was 50.5 months (range, 5 to 82 months), 23 (43%) patients have experienced recurrence, and 15 (28%) have died.

Using targeted massively parallel sequencing, at least 1 somatic mutation was identified in all 54 of the tumor tissues analyzed. We then designed a personalized Safe-SeqS assay for the identified mutation in each patient to quantify ctDNA in a total of 380 serial plasma samples. The median time from date of surgery to postoperative blood collection was 36 days (interquartile range (IQR), 28 to 59 days). ctDNA was detectable in 46 of 54 (85%) patients at baseline (T_0_) and 12 of 49 (24%) patients after surgery (T_P_). For samples with detectable ctDNA, the median ctDNA MAF was 1.86% (IQR, 0.44% to 8.2%) and 0.09% (IQR, 0.02% to 1.3%) at baseline and after surgery, respectively. Clinicopathologic characteristics and their association with baseline and postoperative ctDNA status are shown in Table 1. CEA was elevated in 26 of 46 (57%) patients with detectable ctDNA at baseline and 2 of 12 (17%) patients with detectable ctDNA after surgery. CEA was elevated in 3 of 8 (38%) patients with undetectable ctDNA at baseline and 0 of 36 (0%) patients with undetectable ctDNA after surgery. Clinicopathologic variables significantly associated with postoperative ctDNA detection included the presence of more than 1 liver metastases, a left-sided primary tumor, a node-positive primary tumor, and an elevated postoperative CEA.

**Table 1:** Clinicopathological features, baseline ctDNA, and postoperative ctDNA.

Variable	Baseline ctDNA			Postoperative ctDNA		
	Positive (*N =* 46)	Negative (*N* = 8)	P*	Positive (*N* = 12)	Negative (*N* = 37)	P*
Age, years Median Range	64.8 30.8 to 84.7	51.2 36.4 to 79.4	0.70	56.8 40.5 to 73.5	64.7 30.8 to 84.7	0.80
Sex, no. (%) Female Male	13 (28) 33 (72)	3 (38) 5 (62)	0.60	3 (25) 9 (75)	11 (30) 26 (70)	0.75
Number of liver metastases, no. (%) 1 >1	28 (61) 18 (39)	5 (62) 3 (38)	0.93	4 (33) 8 (67)	25 (68) 12 (32)	**0.036**
Synchronous liver metastases^†^, no. (%) No Yes	24 (52) 22 (48)	5 (62) 3 (38)	0.59	5 (42) 7 (58)	22 (59) 15 (41)	0.28
Time interval from diagnosis of primary tumor to liver metastases, no. (%) <12 months >12 months	27 (59) 19 (41)	5 (62) 3 (38)	0.84	8 (67) 4 (33)	21 (57) 16 (43)	0.54
Primary tumor location, no. (%) Left Right	35 (76) 11 (24)	5 (62) 3 (38)	0.42	12 (100) 0 (0)	25 (68) 12 (32)	**0.023**
Primary tumor N stage, no. (%) N0 N1–2	25 (54) 21 (46)	5 (62) 3 (38)	0.67	1 (8) 11 (92)	26 (70) 11 (30)	**<0.001**
Primary tumor differentiation, no. (%) Well-moderate Poor	41 (89) 5 (11)	7 (88) 1 (12)	0.89	10 (83) 2 (17)	33 (89) 4 (11)	0.59
Baseline CEA elevated (>5 μg/L), no. (%) No Yes	20 (43) 26 (57)	5 (62) 3 (38)	0.32	4 (33) 8 (67)	19 (51) 18 (49)	0.28
Resection margin, no. (%) R0 R1	40 (89) 5 (11)	7 (100) 0 (0)	0.35	11 (92) 1 (8)	32 (91) 3 (9)	0.98
Postoperative CEA elevated (>5 μg/L), no. (%)^‡^ No Yes	39 (95) 2 (5)	7 (100) 0 (0)	0.55	10 (83) 2 (17)	36 (100) 0 (0)	**0.012**

*Bonferroni-adjusted significance threshold = 0.0045.

^†^Synchronous liver metastases = synchronous diagnosis of primary colorectal tumor and metastatic disease.

^‡^CEA was not available in 1 patient with negative postoperative ctDNA.

### ctDNA dynamics during neoadjuvant chemotherapy

**Fig 3:**
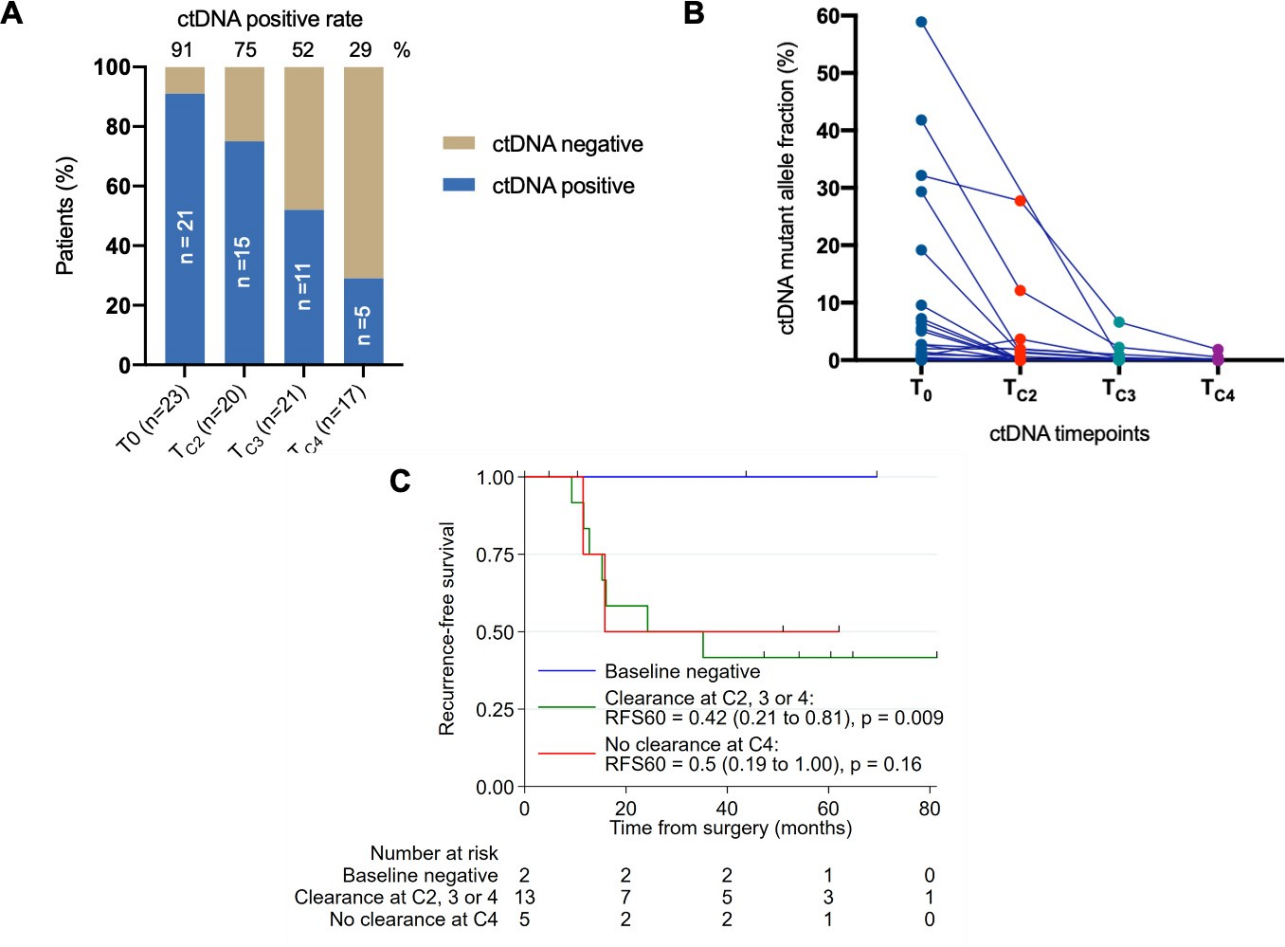
Dynamic changes in ctDNA during neoadjuvant chemo and clinical outcome for cohort 2. (A) ctDNA positive rate at baseline (T_0_) and before cycle 2, 3, and 4 (T_C2_, T_C3_, T_C4_). (B) Changes in ctDNA levels for individual patients before cycle 2, 3, and 4 of neoadjuvant chemotherapy. (C) Recurrence-free survival according to ctDNA clearance. ctDNA, circulating tumor DNA; RFS, recurrence-free survival.

Of the 23 patients in cohort 2 that received neoadjuvant chemotherapy, 21 (91%) were ctDNA positive at baseline. The ctDNA detection rate progressively declined with each cycle of chemotherapy (T_0_ 91%, T_C2_ 75%, T_C3_ 52%, T_C4_ 29%; *P* < 0.001; Fig 3A). On average, there was a 40.93-fold (19.10 to 87.73, *P* < 0.001) decrease in ctDNA level, measured by MAF, during neoadjuvant chemotherapy (mean MAF [SD]: T_0_ 9.9 [16], T_C2_ 0.63 [2.7], T_C3_ 0.11 [0.49], T_C4_ 0.034 [0.13]; Fig 3B). The ctDNA dynamics during neoadjuvant chemotherapy for the individual patients are shown in Fig 3B. Of the 21 patients with positive ctDNA at baseline, 13 had undetectable ctDNA at T_C2_, T_C3_, or T_C4_, and 5 had persistently positive ctDNA at T_C4_. Three cases did not have T_C4_ samples collected and could not be assessed for ctDNA clearance. ctDNA remained undetectable at T_C4_ in the 2 patients with negative ctDNA at baseline and neither had recurred at last follow-up (44 and 69 months from surgery). RFS appeared to be higher in the 2 patients with negative baseline ctDNA than those with positive baseline ctDNA irrespective of ctDNA clearance during neoadjuvant chemotherapy (5-year RFS for T_0_-Negative versus ctDNA clearance at T_C2_, T_C3_, or T_C4_: 100% versus 42%; *P* = 0.009; 5-year RFS for T_0_-Negative versus no ctDNA clearance: 100% versus 50%; *P* = 0.16; Fig 3C), but the study is underpowered to detect a difference due to the small sample size. Comparing baseline and post cycle 4 restaging CT scans, RECIST-defined objective response was observed in 7 of 13 (54%) patients with ctDNA clearance and 4 of 5 (80%) patients with persistently detectable ctDNA at T_C4_ (*P* = 0.596). All 4 patients who achieved a pathological complete response had undetectable ctDNA at T_C3_ or T_C4_.

### ctDNA clearance with adjuvant chemotherapy and clinical outcome

**Fig 4:**
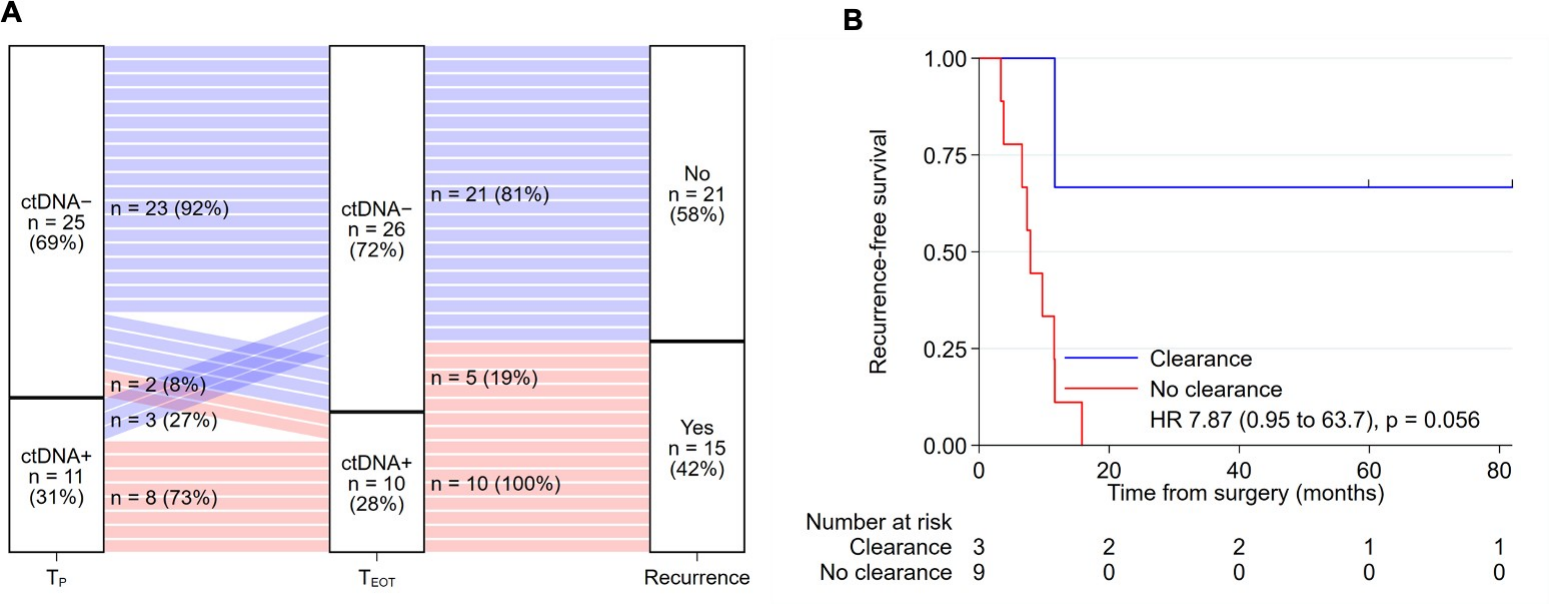
ctDNA dynamics with adjuvant chemotherapy and recurrence. (A) Sankey plot of ctDNA dynamics with adjuvant chemotherapy treatment and recurrence outcome for the 36 patients who had both postoperative (T_P_) and end-of-treatment (T_EOT_) ctDNA samples available for analysis. (B) Recurrence-free survival according to ctDNA clearance after completing adjuvant chemotherapy for patients with positive postoperative ctDNA. ctDNA, circulating tumor DNA.

Of the 42 patients who received adjuvant chemotherapy after surgery, 36 had both postoperative (T_P_) and end-of-treatment (T_EOT_) ctDNA samples available for analysis. Serial T_P_ and T_EOT_ ctDNA status and recurrence outcome for these patients are shown in Fig 4A. For the 11 patients who were T_P_-positive, ctDNA clearance (T_P_-Positive and T_EOT_-Negative) was observed in 3 patients, 2 of whom remained recurrence-free at last follow-up, 60 and 82 months after surgery. All 8 patients who had detectable ctDNA at completion of adjuvant chemotherapy (T_P_-Positive and T_EOT_-Positive) experienced recurrence, with a median time to recurrence of 2.2 months after completion of chemotherapy. The estimated 5-year RFS was 66.7% for patients who cleared their ctDNA after adjuvant chemotherapy compared to 0% in patients with persistently positive ctDNA after adjuvant chemotherapy (HR, 7.87; 95% CI 0.95 to 63.7; *P* = 0.056; Fig 4B). Two patients with an initial negative ctDNA postoperatively developed a positive test at completion of adjuvant chemotherapy (T_P_-Negative and T_EOT_-Positive), and both patients experienced clinical recurrence at 9 and 11 months after completion of chemotherapy.

### Prognostic significance of baseline, postoperative, and end-of-treatment ctDNA

**Fig 5:**
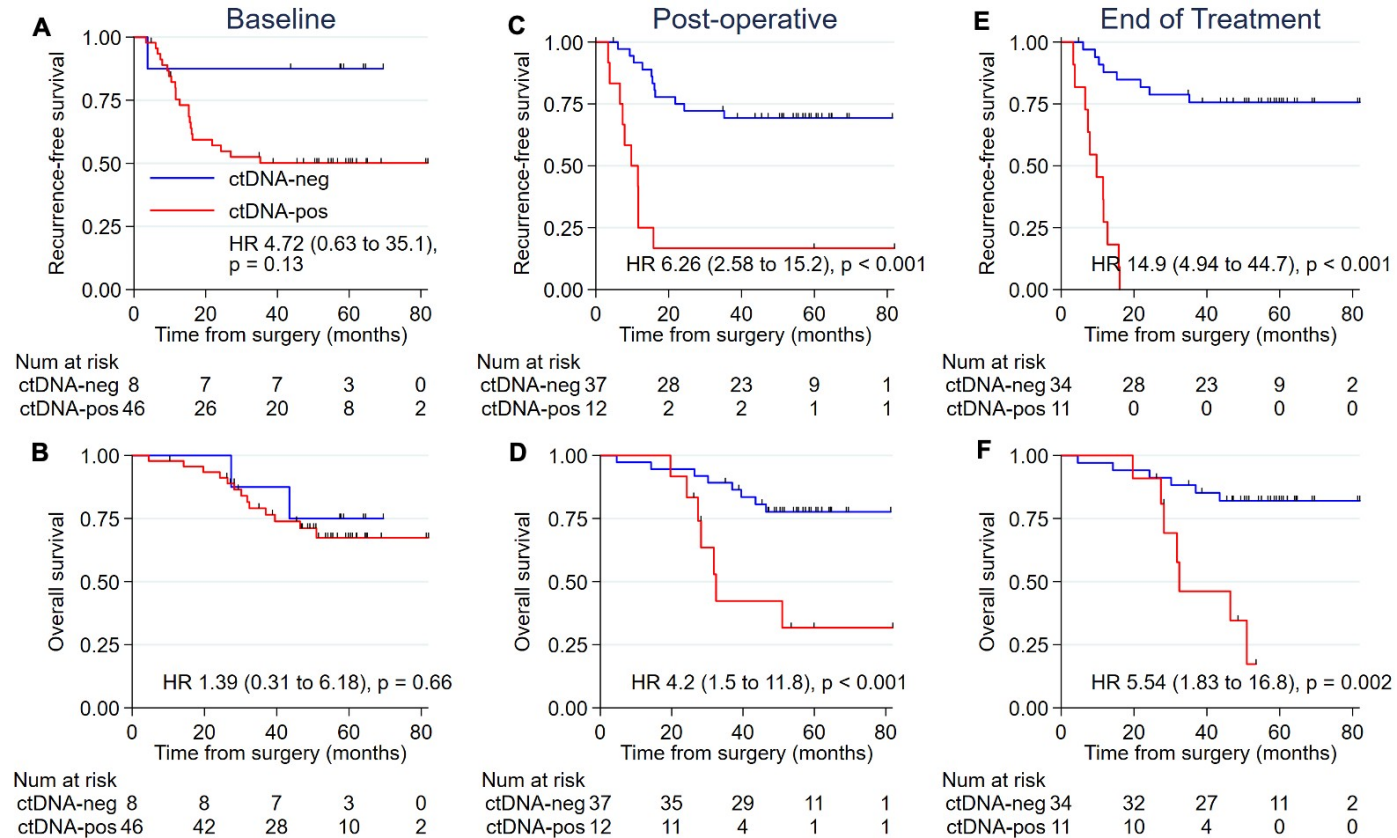
RFS and OS according to ctDNA status at different time points. (A) RFS for baseline ctDNA (T_0_). (B) OS for baseline ctDNA (T_0_). (C) RFS for postoperative ctDNA (T_P_). (D) OS for postoperative ctDNA (T_P_). (E) RFS for end-of-treatment ctDNA (T_EOT_). (F) OS for end-of-treatment ctDNA (T_EOT_). ctDNA, circulating tumor DNA; OS, overall survival; RFS, recurrence-free survival.

We assessed the prognostic impact of baseline, postoperative, and end-of-treatment ctDNA on RFS and overall survival (OS). A nonsignificant trend for worse RFS was observed for patients with a positive compared to negative baseline ctDNA (Fig 5A), but no difference was seen for OS (Fig 5B). Patients with a positive ctDNA status after surgery or at the end of all treatment had a significantly lower RFS and OS compared to those with negative ctDNA at these time points (Fig 5C–5F). The Kaplan–Meier estimates of RFS at 5 years were 16.7% and 69.3% for the postoperative ctDNA-positive and ctDNA-negative groups (HR, 6.26, 95% CI 2.58 to 15.2; *P* < 0.001), and 0% and 75.6% for the end-of-treatment ctDNA-positive and ctDNA-negative groups (HR, 14.9, 95% CI 4.94 to 44.7; *P* < 0.001). Five-year OS estimates were 31.7% and 77.7% for the postoperative ctDNA-positive and ctDNA-negative groups (HR, 4.2, 95% CI 1.5 to 11.8; *P* < 0.001), and 17.3% and 82.0% for the end-of-treatment ctDNA-positive and ctDNA-negative groups (HR, 5.54, 95% CI 1.83 to 16.8; *P* = 0.002).

In our study population, primary tumor N stage was the only clinicopathologic variable found to be significantly associated with RFS in univariate analysis (Table 2). A trend for lower RFS was observed in patients with an elevated baseline CEA. To adjust for multiple variables in a single model, we used a Cox proportional hazard model. Postoperative ctDNA status remained an independent predictor of RFS (HR, 3.13; 95% CI 1.00 to 9.82, *P* = 0.050; Table 2), along with primary tumor N stage. Baseline CEA was a significant predictor of RFS on multivariate analysis. We did not observe a significant difference in postoperative ctDNA detection rate and sites of relapse. Of the 10 cases with liver recurrences, 5 (50%) had positive postoperative ctDNA; of the 11 cases with extrahepatic disease only, 5 (45%) had positive postoperative ctDNA.

**Table 2:** Univariate and multivariate Cox regression for recurrence-free survival.

Outcome	Univariate		Multivariate	
	HR (95% CI)	P*	HR (95% CI)	P*
Postoperative ctDNA (negative vs positive)	6.31 (2.59 to 15.37)	**<0.001**	3.13 (1.00 to 9.82)	**0.050**
Baseline CEA (not elevated vs elevated)	2.32 (0.95 to 5.66)	0.064	3.30 (1.17 to 9.33)	**0.024**
Number of liver metastases (1 vs >1)	1.34 (0.59 to 3.07)	0.49	0.37 (0.12 to 1.12)	0.078
Diameter of largest liver metastasis >3 cm (no vs yes)^†^	1.77 (0.78 to 4.02)	0.18	1.59 (0.61 to 4.16)	0.34
Time interval from diagnosis of primary tumor to liver metastases (<12 months vs >12 months)^‡^	0.65 (0.28 to 1.54)	0.33	0.40 (0.14 to 1.12)	0.080
Primary tumor N stage (N0 vs N+)	4.39 (1.84 to 10.49)	**0.001**	10.72 (2.72 to 42.29)	**0.001**

*Bonferroni-adjusted significance threshold = 0.012.

^†^Reference [21].

^‡^Reference [22].

CEA, carcinoembryonic antigen; ctDNA, circulating tumor DNA.

### Pre- and postoperative ctDNA, and ctDNA during surveillance

**Fig 6:**
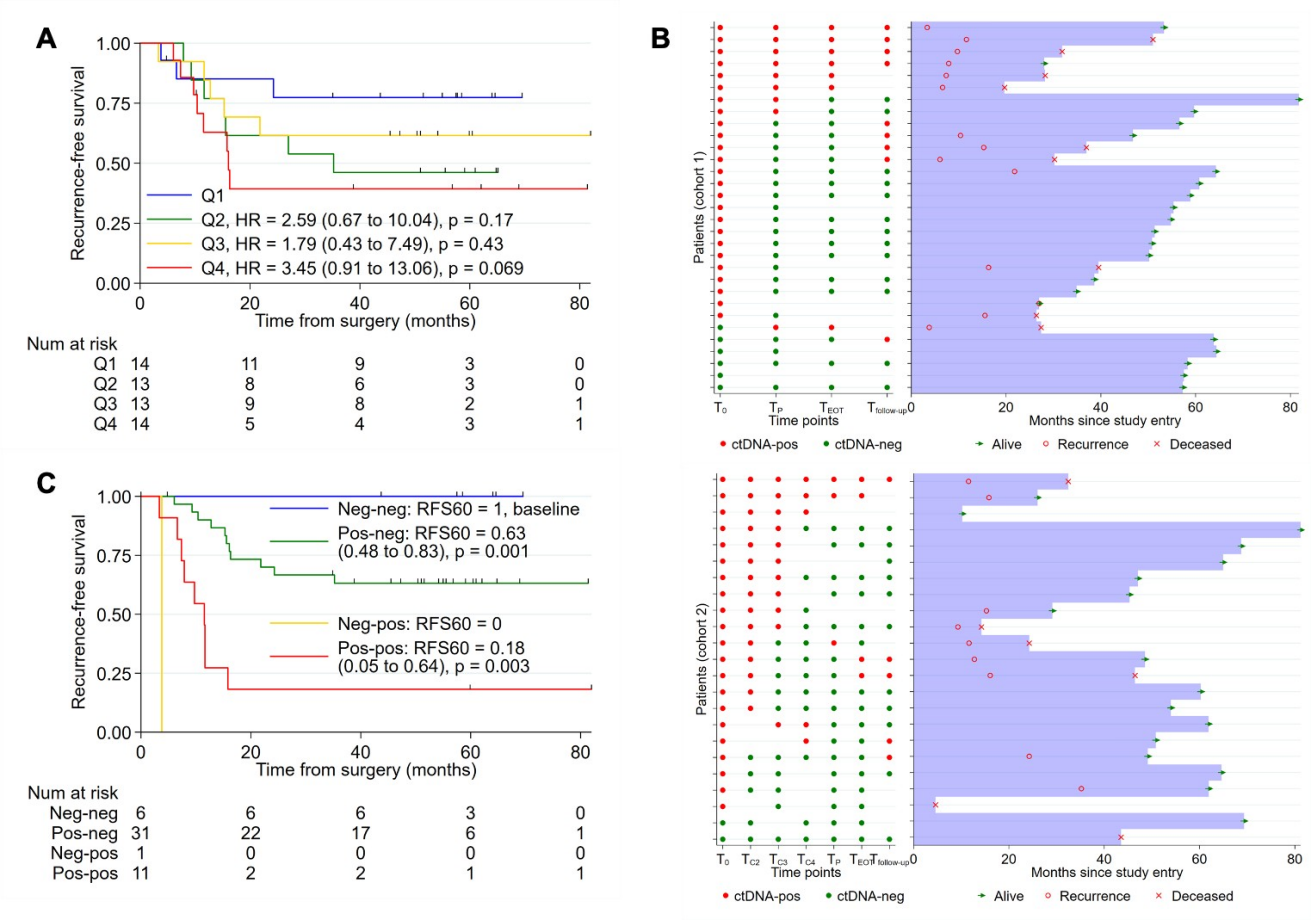
Baseline ctDNA MAF, pre- and postoperative ctDNA, and clinical outcome. (A) RFS stratified by baseline ctDNA MAF quartiles; MAF cut-offs: Q1 ≤ 0.15, Q2 > 0.15 and ≤ 1.04, Q3 > 1.04 and ≤ 7.2, Q4 > 7.2. (B) Swimmer plot showing serial ctDNA detectability and clinical outcomes for patients in cohort 1 and 2. (C) RFS according to serial baseline and postoperative ctDNA status. ctDNA, circulating tumor DNA; MAF, mutant allele fraction; RFS, recurrence-free survival.

RFS estimates stratified by baseline ctDNA MAF quartiles are shown in Fig 6A. There was a trend for a worse RFS for patient with the highest quartile of MAF compared to patients with the lowest quartile of MAF (HR 3.45, 95% CI 0.91 to 13.06, *P* = 0.069). A swimmer plot of serial ctDNA detectability and clinical outcomes for cohort 1 and 2 patients is shown in Fig 6B. Of the 49 patients with both baseline (T_0_) and postoperative (T_P_) ctDNA samples, 6 (12%) were negative at both time points, 1 (2%) was T_0_-Negative and T_P_-Positive, 31 (63%) were T_0_-Positive and T_P_-Negative, and 11 were (22%) positive at both time points. None of the patients with negative ctDNA results at both time points have recurred. The 5-year RFS estimates based on T_0_ and T_P_ ctDNA detections are: 100% for T_0_-Negative and T_P_-Negative, 63% for T_0_-Positive and T_P_-Negative, 18% for T_P_-Positive and T_P_-Positive, and 0% for T_0_-Negative and T_P_-Positive (Fig 6C). Sixteen of the 23 patients with recurrence had blood samples collected prior to or at the time of recurrence, with ctDNA being detected in 14 of 16 (87.5%) cases.

## Discussion

For patients with resectable CRLM, there are currently no biomarkers with proven clinical utility in the personalization of perioperative or adjuvant systemic therapy. We report here the largest study to our knowledge with comprehensive serial ctDNA profiling before and after resection of CRLM, demonstrating the potential of serial sampling as a real-time marker of neoadjuvant and postoperative chemotherapy impact in this setting.

Complete surgical resection of all disease, in combination with oxaliplatin-based chemotherapy, is the current gold standard for management of resectable CRLM [2]. Multiple prognostic systems have been developed for this patient group, with the number of liver metastases, the stage of the primary tumor, the size of the largest metastasis, the preoperative CEA level, and the presence of extrahepatic metastases, the factors considered of highest predictive value [21,23]. While these factors alone or in combination can be used to estimate the likelihood of recurrent disease, with the exception of extrahepatic metastases, they are not routinely used to inform individual patient management. A biomarker that more precisely estimates recurrence risk could be used to guide initial perioperative and adjuvant therapy decision-making as well as the optimal surveillance strategy for individual patients. Serial sampling of such a biomarker and observed changes over time could further inform patient management.

The prognostic significance of ctDNA analysis following hepatic resection in our series of patients reproduces the findings in the initial small series of patients with resected CRLM [14]. In this earlier study, 15 of 16 patients (93.8%) with detectable ctDNA after surgery had disease recurrence, whereas in the current series, 10 of 12 (83%) such patients have recurred. Notably, the 2 patients who did not recur did not have detectable ctDNA at the completion of adjuvant therapy, consistent with chemotherapy having eradicated the minimal residual disease present after surgery that was the initial source of the ctDNA and the potential source of later disease recurrence. For patients with negative ctDNA postsurgery, there appears to be a low risk of recurrence (30% in the current series and 0% in the earlier series), suggesting a shorter duration chemotherapy or even no adjuvant therapy should be explored as a treatment strategy given the likely minimal impact of treatment in this patient subset. Intriguingly, when considering both preoperative (baseline) and postoperative ctDNA, none of the 6 patients who had undetectable ctDNA at both time points experienced recurrence, suggesting stratification by preoperative assessment of ctDNA could add further prognostic information to the postoperative ctDNA analysis. If the favourable prognostic impact of having negative pre- and postoperative ctDNA is validated, this may be a subgroup of patients where adjuvant chemotherapy may be shortened or omitted.

Consistent with the findings from our previous studies in early stage colon cancer [16,24], this study confirms the possibility that serial ctDNA analysis is a potential real-time marker of adjuvant therapy impact, with ctDNA clearance providing an early measure of the effectiveness of adjuvant treatment. Alternatively, given the 100% recurrence risk in patients where ctDNA is persistently detectable after adjuvant chemotherapy, this is a patient group where the value of further therapy with novel systemic strategies or more intensive surveillance should be explored in clinical trials. ctDNA clearance rate could also be used as a “go/no-go” decision guide earlier in studies exploring new adjuvant therapy approaches for patients with resectable CRLM, as well as a surrogate endpoint for conventional trial endpoints such as RFS and OS following further validation.

We have previously demonstrated that reduction in ctDNA levels following a single cycle of systemic treatment is associated with tumor response on first restaging imaging (after 4 cycles of chemotherapy) in patients with metastatic CRC being treated with palliative intent [11]. In the current cohort of patients with upfront resectable CRLM, we observed a rapid decline in ctDNA in the majority of patients treated with neoadjuvant chemotherapy with 72% of those testing positive at baseline experienced ctDNA clearance by cycle 2, 3, or 4. While this early clearance of ctDNA was not associated with a better RFS compared to patients who have persistently detectable ctDNA at cycle 4 of chemotherapy, the modest sample size may have limited our ability to detect such an association.

There are several limitations to our study. The sample size was small, the potential for false-positive findings with multiple hypothesis testing, and a range of systemic therapy approaches were utilized. Of note, a major challenge with ctDNA detection of minimal residual disease in solid tumors is the analytical sensitivity of the ctDNA assays, in other words, the false negative results. In this and our previous studies in nonmetastatic CRC [16,18,24], we have found that using a 15-gene panel is sufficient to detect at least 1 clonal mutation in virtually all colorectal tumor tissue, which then allows us to track at least one of these mutations in the plasma at all time points analyzed. While this method is highly specific in predicting recurrence, we found that 5 of the 15 patients who experienced recurrence tested negative at the end of all treatment (Fig 4B). How to increase assay sensitivity is an important question to address in future studies. One potential strategy would be to increase the volume of plasma, another way would be to assess more mutations, as does the Signatera assay which involves whole exome sequencing of the tumor tissue and then interrogates up to 16 variants in the plasma. However, to identify more mutations requires >100 times more sequencing of the primary tumor DNA, ordering personalized primer pairs for every patient, and more sequencing of plasma DNA, which substantially increases cost. Moreover, the more mutations analyzed, the more artefactual mutations are detected, compromising assay specificity. A more focussed panel such as our 15-gene panel has several advantages over genome-wide or exome-wide screening for ctDNA detection, including lower cost (approximately $1 per patient for a panel of primers) and increased specificity. Even with advances in ctDNA assay, false negative results can still occur due to biological factors such as low DNA shedding tumours, mucinous histology, and anatomical location of the occult micrometastatic disease. Ultimately, the optimal approach will have to balance cost, throughput, and specificity, and different approaches may be most suitable for patients depending on the type of cancer, disease stage, and the clinical application. Most importantly, prospective studies where the ctDNA results are used to inform patient management are required to define the value of a ctDNA-guided approach to patient management.

In summary, we have confirmed the prognostic significance of detecting ctDNA at defined time points in patients undergoing resection for CRLM and, for the first time, demonstrated the potential value of serial analysis during adjuvant therapy and during surveillance in such patients. Further studies of ctDNA in this population are required to demonstrate the clinical utility of a ctDNA-informed approach to treatment and surveillance strategies, including the ultimate impact on recurrence-free and overall survival.

## Supporting information

S1 REMARK Checklist REporting recommendations for tumour MARKer prognostic studies (REMARK). (DOCX)

## Abbreviations

CEA: carcinoembryonic antigen
cfDNA: cell-free DNA
CRC: colorectal cancer
CRLM: colorectal cancer liver metastases
ctDNA: circulating tumor DNA
IQR: interquartile range
MAF: mutant allele fraction
OS: overall survival
RFS: recurrence-free survival
Safe-SeqS: Safe-Sequencing
